# Serum amyloid P component accumulates and persists in neurones following traumatic brain injury

**DOI:** 10.1098/rsob.230253

**Published:** 2023-12-06

**Authors:** Ping K. Yip, Zhou-Hao Liu, Shumaila Hasan, Mark B. Pepys, Christopher E. G. Uff

**Affiliations:** ^1^ Centre for Neuroscience, Surgery & Trauma, Blizard Institute, Barts and The London School of Medicine and Dentistry, Queen Mary University of London, London E1 2AT, UK; ^2^ Department of Neurosurgery, Chang Gung Memorial Hospital at Linkou, Chang Gung University, Taoyuan 33302, Taiwan; ^3^ Department of Neurosurgery, Royal London Hospital, Whitechapel, London E1 1FR, UK; ^4^ Wolfson Drug Discovery Unit, University College London, London NW3 2PG, UK

**Keywords:** traumatic brain injury, serum amyloid P component, neurodegeneration, dementia

## Abstract

The mechanisms underlying neurodegenerative sequelae of traumatic brain injury (TBI) are poorly understood. The normal plasma protein, serum amyloid P component (SAP), which is normally rigorously excluded from the brain, is directly neurocytotoxic for cerebral neurones and also binds to A*β* amyloid fibrils and neurofibrillary tangles, promoting formation and persistence of A*β* fibrils. Increased brain exposure to SAP is common to many risk factors for dementia, including TBI, and dementia at death in the elderly is significantly associated with neocortical SAP content. Here, in 18 of 30 severe TBI cases, we report immunohistochemical staining for SAP in contused brain tissue with blood–brain barrier disruption. The SAP was localized to neurofilaments in a subset of neurones and their processes, particularly damaged axons and cell bodies, and was present regardless of the time after injury. No SAP was detected on astrocytes, microglia, cerebral capillaries or serotoninergic neurones and was absent from undamaged brain. C-reactive protein, the control plasma protein most closely similar to SAP, was only detected within capillary lumina. The appearance of neurocytotoxic SAP in the brain after TBI, and its persistent, selective deposition in cerebral neurones, are consistent with a potential contribution to subsequent neurodegeneration.

## Graphical abstract

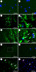

## 1. Introduction

Traumatic brain injury (TBI) is the most prevalent common neurological disorder [1] and its incidence is increasing [2]. A 2012 European Union study found that TBI comprised 37% of all injury related deaths with 87 000 deaths in more than 2 million TBI admissions [3], and it causes most deaths and disability below age 40 in the developed world [4]. The economic cost of TBI, which includes set-up and maintenance of the infrastructure and pathways for the acute and long-term management of patients [5], was estimated at £5 billion per year in the UK [6], and is currently estimated at US$400 billion globally [1], an increase of 33% from 2017 [7]. A 2014 Taiwanese study demonstrated a 10.9% decrease in mortality over 12 years from 1998 to 2010, associated with a 47.6% increase in the proportion of surgical interventions and a 19% increase in the mean hospital treatment cost [8]. One large cohort study showed a greater than 40% cumulative mortality rate at 13 years after all types of head injury [9] and the highly variable outcome in survivors [10] was affected by age, handedness, pre-existing medical conditions, anatomical variants, history of TBI and genetic predisposition [11]. The social impact is immense: A total of 50% of homeless people in one study reported previous significant TBI, 90% of which occurred before they became homeless [12]. The very high prevalence of past TBI in the UK prison population, with the resulting behavioural problems, is likely a significant contributor to reoffending [13].

There is compelling evidence that TBI can trigger neurodegeneration [14], that age-related cerebral atrophy is accelerated after TBI [15], and that this is a major determinant of long-term outcome [16]. Single or repetitive mild TBI can lead to dementia with an increased hazard ratio of 2.4 if there is no loss of consciousness and 2.5 with loss of consciousness, increasing to 3.8 for moderate and severe TBI [17]. Even mild TBI can be associated with chronic traumatic encephalopathy [18] and a single significant injury can predispose individuals to late cognitive decline and Alzheimer's disease [19–21]. The cerebral amyloid deposits composed of A*β* protein that are a key neuropathological feature of Alzheimer's disease (AD) are also a notable feature of chronic traumatic encephalopathy [22], appearing in the brain almost immediately after TBI and persisting long-term [16,23]. However, the mechanisms that are directly responsible for post-traumatic neurodegeneration are poorly understood.

Serum amyloid P component (SAP) is an invariant, constitutive normal plasma glycoprotein produced exclusively in the liver. It circulates at a mean (s.d.) concentration of about 24 (8) mg l^−1^ in women and 32 (7) mg l^−1^ in men [24], but it is normally rigorously excluded from the CNS. Cerebrospinal fluid (CSF) concentrations of SAP are 1000-fold lower than the plasma concentrations [25,26], presumably reflecting impermeability of the blood–brain barrier (BBB) and there is also evidence for an active transport mechanism exporting SAP from the CSF back into the blood [27]. SAP is named for its universal presence in all human amyloid deposits, which reflects the avid but reversible calcium dependent binding of SAP to all types of amyloid fibrils regardless of their protein composition [28,29]. Thus, although CSF and brain content of SAP are normally extremely low, SAP is nonetheless always present in AD on intracerebral A*β* amyloid plaques, cerebrovascular A*β* amyloid deposits and the majority of neurofibrillary tangles. The binding of SAP stabilizes amyloid fibrils [30] and promotes their formation [31,32], thereby contributing to both amyloid deposition and persistence [33].

However, although cerebral A*β* amyloid is usually present in chronic traumatic encephalopathy and always in AD, it is still not known whether and how amyloid pathology contributes to the neurodegeneration that underlies cognitive loss, although cognitive benefit from antibody treatments that reduce the A*β* amyloid burden in AD has lately been reported [34,35]. The fact that human SAP itself, unrelated to its roles in amyloid formation and persistence, is directly neurotoxic to cerebral neurones both *in vitro* [36–38] and in animal models *in vivo* [39], is therefore important. The recent demonstration, in the cognitive function and ageing study of an unselected, population-representative cohort, that neocortical SAP content is significantly associated with cognitive status at death, independent of Braak stages, Thal phase and all other classical neuropathological hallmarks of dementia [40] is consistent with SAP in the brain contributing directly to neurodegeneration.

Frank blood in the brain after TBI must inevitably increase exposure of the brain to SAP but we have recently demonstrated histologically that there is also more subtle disruption of the BBB in the acute phase following TBI [41]. Here we report for the first time, that immunofluorescence staining of brain tissues from TBI patients, detects SAP in contused and adjacent cerebral tissue, specifically localized to neurofilaments within a subset of neuronal cell bodies and their processes. These observations are consistent with the known acute neurotoxicity of SAP participating in the neurodegeneration responsible for cognitive sequelae of TBI.

## 2. Material and methods

### 2.1. TBI brain samples

The Royal London Hospital (London, UK) and Chang Gung Memorial Hospital (Taoyuan, Taiwan) recruited patients suffering severe TBI where brain tissue was sampled opportunistically if the dura was breeched for therapeutic or diagnostic purposes. Thus, brain tissue that was either severely contused and surgically resected, or that had been traumatically displaced by ballistic or penetrating injury, was retained for research. In addition, the superior frontal gyrus was biopsied prior to inserting an intracranial pressure (ICP) monitor or an external ventricular drain (EVD). All approvals and tissue collection methods are reported in the severe head injury brain analysis (SHIBA) study [41], which was supported by a significant public engagement project to define research questions and protocols in TBI [42]. Table 1 summarizes tissue collection timings and characteristics. All brain tissue was fixed in 10% neutral buffered formalin for at least 2 h. A small sample was then frozen in dry ice and stored at −80°C. The rest of each sample was cryoprotected in 20% w/v sucrose and stored at 4°C. TBI brain samples were also similarly collected in Taiwan (table 1), with local ethical approval. These samples were snap frozen without fixation and stored in liquid nitrogen.

**Table 1.** Immunostaining for serum amyloid P component in brain tissue after severe traumatic brain injury. SHIBA, severe head injury brain analysis [41].

SHIBA case no	tissue type	collection time after injury (h)	SAP detected	tissue collection
1	biopsy	147	Yes	craniotomy
2	biopsy	6	No	ICP monitor insertion
3	biopsy	3.5	No	ICP monitor insertion
3	traumatic	24	Yes	traumatically displaced
4	biopsy	8	No	ICP monitor insertion
5	biopsy	2	No	ICP monitor insertion
6	biopsy	75	No	ICP monitor insertion
6	contused	97	Yes	craniotomy
6	contused	243	Yes	craniotomy
7	biopsy	8	Yes	craniotomy
8	biopsy	4	No	ICP monitor insertion
9	biopsy	4	No	ICP monitor insertion
10	biopsy	9	No	EVD insertion
10	traumatic	96	Yes	EVD insertion
11	biopsy	2	No	ICP monitor insertion
12	biopsy	2	No	craniotomy
12	resection	2	Yes	craniotomy
13	biopsy	46	No	craniotomy
14	biopsy	19	Yes	craniotomy
14	resection	204	Yes	craniotomy
15	biopsy	3	Yes	EVD insertion
16	biopsy	5	No	ICP monitor insertion
17	biopsy	17	Yes	craniotomy
18	biopsy	5	No	craniotomy
18	resection	432	No	craniotomy
19	biopsy	3	Yes	craniotomy
19	resection	2.5	Yes	craniotomy
20	biopsy	5	Yes	ICP monitor insertion
21	biopsy	3	Yes	craniotomy
21	resection	4	Yes	craniotomy
21	resection	147	Yes	craniotomy
22	biopsy	22	No	ICP monitor insertion
23	biopsy	4	No	ICP monitor insertion
23	traumatic	8	Yes	traumatically displaced
24	biopsy	5	No	craniotomy
25	biopsy	4	No	craniotomy
**Taiwan case no**				
99	resection	42	Yes	craniotomy
61	resection	4	Yes	craniotomy
13	resection	3	Yes	craniotomy
40	resection	2	Yes	craniotomy
90	resection	8	Yes	craniotomy

### 2.2. Immunofluorescence

As previously described [41,43], 10 µm cryosections of small fragments of fixed brain tissue stored at 4°C and at −80°C were thaw-mounted onto microscope slides. Sections of tissue stored at −80°C or in liquid nitrogen were fixed with 4% w/v paraformaldehyde for 5 min before immunostaining. In contrast to the UK tissues, the tissues harvested and stained in Taiwan had been snap frozen without any prior fixation. All slides were washed three times with PBS for 5 min each before incubation for 30 min with antigen unmasking solution (Vector Laboratories Cat. No. H-3300) pre-heated at 80°C, followed by cooling at room temperature for 10 min and a further three 5 min washes with PBS. Non-specific antibody binding to the sections was then blocked by incubation for at least 30 min with either 2% w/v skimmed milk solution (UK) or 10% v/v normal donkey serum (Taiwan). The various primary antibodies at the concentrations shown in electronic supplementary material, table SI were placed on the sections and incubated overnight at room temperature before washing by immersion in PBS for three 5 min periods. Bound primary antibodies were then detected by staining for 2 h at room temperature with the fluorescent conjugated secondary antibodies shown in electronic supplementary material, table SI. The sections were finally incubated with Hoechst 3342 at 1 : 100 for 5 min to counterstain the nuclei before repeating the PBS wash cycle and mounting in VectaShield medium (Vector Laboratories cat. no. H-1000). For staining with anti-C-reactive protein (CRP) antibodies, PBS was replaced throughout with 140 mM NaCl, 2 mM CaCl_2_, 10 mM Tris, pH 8.0 to prevent disruption of the calcium dependent specific ligand binding of CRP and to stabilize the native CRP pentamer structure.

Immunospecificity of the staining for SAP was confirmed by preabsorption of the monoclonal mouse anti-human SAP antibody, SAP-5, with an excess of isolated pure human SAP [44]. This completely abolished the positive staining observed on serial sections of the same tissue samples stained with the unabsorbed anti-SAP antibody. Specificity of staining with anti-CRP was similarly confirmed by preabsorption with isolated pure human CRP [44].

Images of the immunostained sections were recorded in the UK at ×40 magnification using a Zeiss Axioskop 2 fluorescent microscope with a Hamamatsu CCD digital camera and HiPic v9.1 software. The images in Taiwan were recorded at ×40 magnification using a Zeiss Axioskop 4 fluorescent microscope with a Forever Plus microscope camera and the FPC-EE2M camera system. For display, images were pseudo-coloured and overlaid using Adobe Photoshop.

## 3. Results

### 3.1. Immunodetection of SAP in injured brain tissue

**Figure 1.**
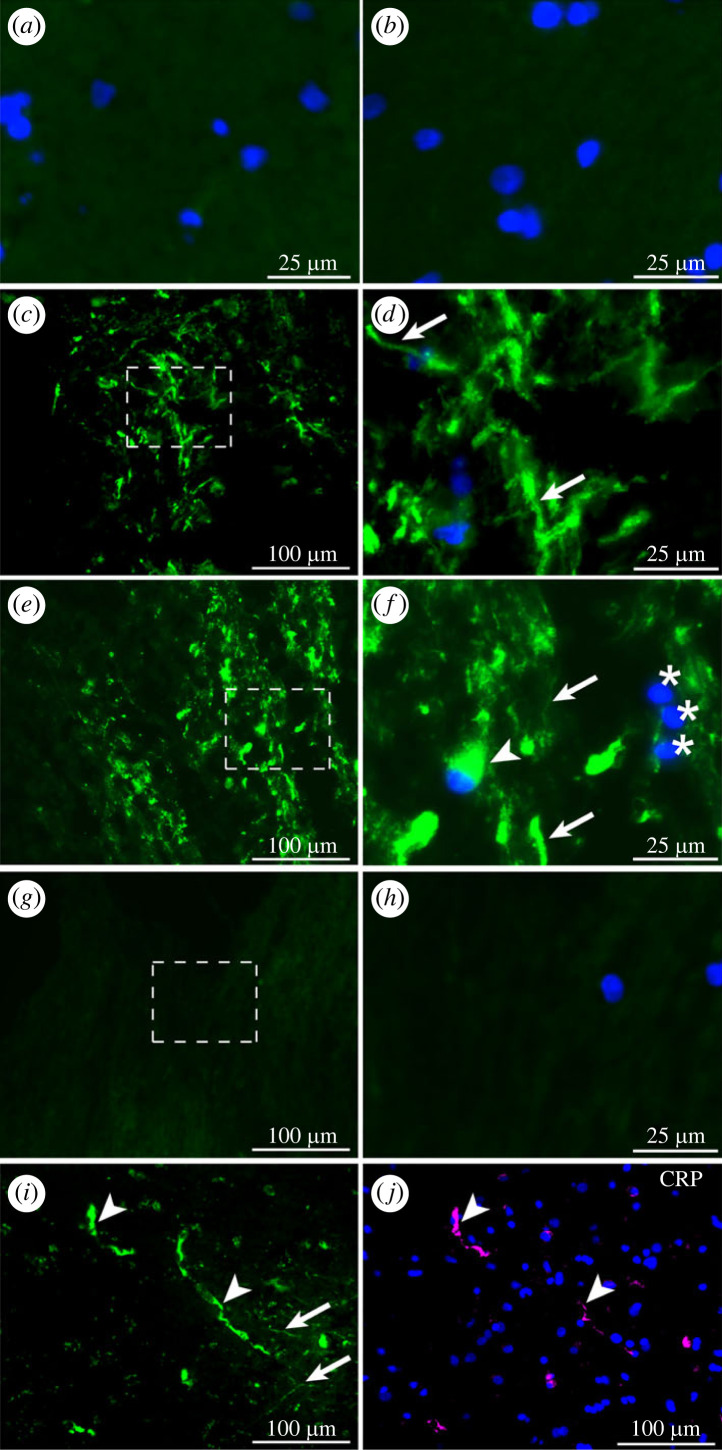
Immunostaining for SAP in brain tissue after TBI. (*a,b*) No immunostaining for SAP was observed in sections of uninjured brain obtained by biopsy of the left (*a*) or right (*b*) superior frontal gyrus. (*c–f*) Strong positive immunostaining for SAP was present in some cell bodies (arrowhead) and processes (arrows) in sections of contused brain after severe TBI; other cell bodies were negative (asterisks). (*g,h*) Preabsorption of the primary anti-SAP antibody with isolated pure human SAP completely inhibited the immunostaining. (*d,f,h*) Enlarged images of the dashed boxes in *c, e, g*, respectively. Double immunostaining of the same sections with anti-SAP (*i*) and anti-CRP (*j*) antibodies showed distinctly different patterns, with CRP detected only within the lumina of blood vessels (arrowheads) and not in the parenchyma or associated with neurones. Scale bars: *a, b, d, f, h*: 25 µm; *c, e, g, i, j*: 100 µm. Some images include blue nuclear counterstaining with Hoechst.

SAP was not detected in sections of superior frontal gyrus tissue obtained at ICP monitor or EVD insertion or of other brain regions without macroscopic signs of injury (figure 1*a,b*). However, there was definite immunostaining for SAP in sections from 13 of the 25 severe TBI patients in the SHIBA study (figure 1*c–f*).[41] Among these cases, SAP was detected in 8 of the 9 macroscopically contused brain tissues with evident BBB disruption. BBB disruption was established by detection of frank blood on CT scan of the affected region, by macroscopic observation of contusion with petechial haemorrhages, and/or by microscopic demonstration of significant disruption of the anti-claudin 5 and anti-von Willebrand Factor (vWF) immunostaining of the microvascular architecture. The predominant strand-like morphology of SAP staining was consistent with SAP location on or in neuronal processes. In addition, there was clear immunostaining for SAP within a minor subset of neuronal cell bodies (figure 1*f*). SAP antibody that had been preabsorbed with pure human SAP produced no staining (figure 1*g,h*), confirming the immunospecificity of the positive results.

The immunoreactivity of human SAP is markedly reduced by standard formalin fixation of tissues [45]. Positive immunostaining after antigen retrieval in sections of formalin fixed tissues, with immunospecificity confirmed by specific antigen absorption, therefore robustly demonstrated the presence of SAP in the tissues. Furthermore, the tissues studied in Taiwan that were snap frozen without any prior fixation, ensuring full retention of SAP antigenic reactivity, showed the same positive staining pattern for SAP as in the UK series. On the other hand, absence of immunostaining for SAP in a proportion of the UK cases does not exclude the presence of SAP but may just reflect fixation damage.

The immunodetection of SAP within contused brain tissue did not simply reflect non-specific entry of circulating plasma proteins. Double immunostaining for CRP, which is closely related and extremely similar to SAP in all respects except their calcium dependent ligand binding specificity [46], was positive only within vascular lumina, not in the brain tissue itself (figure 1*i,j*). Importantly, this notably different distribution of CRP was present regardless of the time the tissue was taken after injury and of the varying serum concentrations of CRP, which overlapped and, in many cases, greatly exceeded the normal, constitutive concentrations of SAP [24] (electronic supplementary material, table SII).

### 3.2. Axonal location of SAP deposition

**Figure 2.**
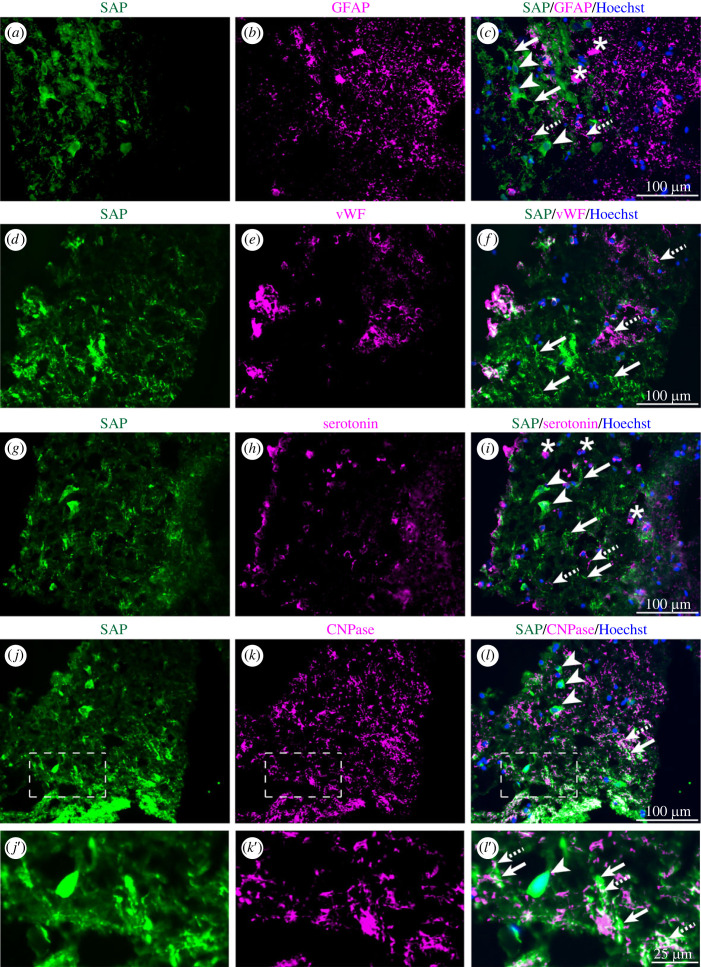
Axonal deposition of SAP in contused brain tissue after TBI. Double immunostaining of sections with anti-SAP and other antibodies. (*a–c*) Immunostaining for SAP in cell processes (arrows) and cell bodies (arrowheads) was not colocalized with immunostaining for GFAP in astrocytic processes (dashed arrow) and cell bodies (asterisks). (*d–f*) Immunostaining for SAP (arrows) was rarely in close proximity to immunostaining for the vWF capillary endothelial cell marker (dashed arrows). (*g–i*) Immunostaining of cell processes (arrows) and cell bodies (arrowheads) for SAP did not co-localize with immunostaining for serotonin in fibres (dashed arrows) or cell bodies (asterisks). (*j–l*′) SAP immunostaining in processes (arrows) was often colocalized with immunostaining for the CNPase myelin marker (dashed arrows) but this colocalization was not seen in cell bodies which lack CNPase (arrowhead). (*a, d, g, j, j*′) SAP immunostaining in green. (*b, e, h, k, k*′) Other immunostained targets in magenta. (*c, f, i, l, l*′) Merged adjacent images to identify colocalization. (*j*′–*l*′) Enlargements of dashed box regions of adjacent images. Scale bars: *c, f, i, l*: 100 µm; L': 25 µm. Some images include blue nuclear counterstaining with Hoechst.

Immunostaining for SAP did not colocalize with the astrocyte-specific antigen, glial fibrillary acid protein (GFAP) (figure 2*a–c*), with the endothelial cell marker, vWF (figure 2*d–f*), or with serotonin, the marker of serotoninergic fibres (figure 2*g–i*). CNPase, the myelin-specific antigen known to be absent from cell bodies, was not detected on SAP-positive neuronal cell bodies but it was present in close proximity to SAP-positive processes (figure 2*j–l*), identifying these as myelinated axons.

### 3.3. SAP is deposited on neurofilaments

**Figure 3.**
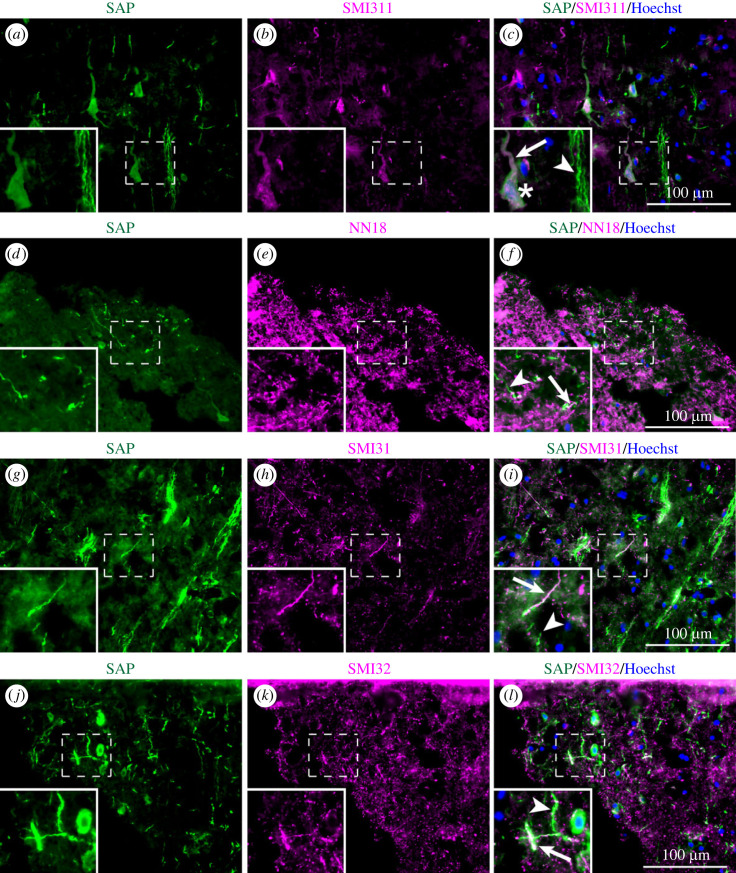
Colocalization of SAP and some neurofilaments in contused brain tissue after TBI. Double immunostaining of single sections for SAP is shown in green and for various neurofilament markers in magenta. In each image of SAP immunostaining, colocalization is indicated by arrows and SAP staining of structures not reactive with anti-neurofilament antibodies is indicated by arrowheads. (*a–c*) Staining by SMI311, an antibody mixture that recognizes non-phosphorylated neurofilament heavy (200 kDa) and neurofilament medium (160 kDa) subunits, colocalized with some SAP immunostaining in neuronal cell body (asterisks) and apical dendrites (arrow). (*d–f*) Immunostaining with NN18, which recognizes non-phosphorylated neurofilament medium (160 kDa) subunits, colocalized with some SAP immunostaining. (*g–i*) Immunostaining with SMI31, which recognizes phosphorylated neurofilament heavy (200 kDa), colocalized with some SAP immunostaining. (*j–l*) Immunostaining with SMI32, which recognizes non-phosphorylated neurofilament heavy (200 kDa), colocalized with some SAP immunostaining. Inserts are enlarged images of the dashed boxes. Scale bars: 100 µm. Some images include blue nuclear counterstaining with Hoechst.

Deposition of SAP on the variably phosphorylated neurofilament components of the neuronal cytoplasmic, dendritic and axonal cytoskeleton was demonstrated by double immunostaining with anti-SAP and various different anti-neurofilament antibodies. Staining with SMI311, a mixture of antibodies that recognizes non-phosphorylated heavy and medium neurofilament subunits (NF-H and NF-M) [47], identified these proteins as sites of SAP deposition in some neuronal bodies and apical dendrites (figure 3*a–c*). Double immunostaining of SAP positive brain sections with NN18 antibody, which specifically recognizes the non-phosphorylated NF-M subunit [48], confirmed this localization in some but not all axons (figure 3*d–f*). Similarly, double immunostaining with anti-SAP and with SMI31 and SMI32 antibodies, which recognize phosphorylated and non-phosphorylated NF-H, respectively [49], showed SAP deposition on neurofilaments in some axons (figure 3*g–l*).

### 3.4. SAP deposition on NF200 immunopositive structures

**Figure 4.**
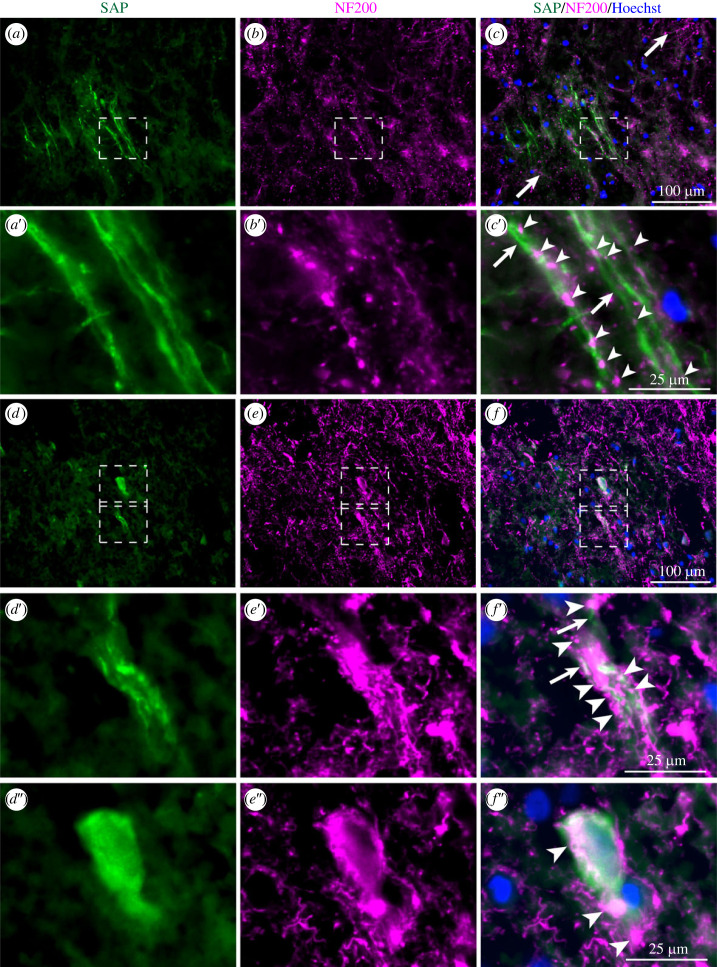
Colocalization of SAP and NF200 in damaged axons. Double immunostaining of single sections of contused brain for SAP (green in *a*, *d*) and NF200 (magenta in *b*, *e*), and superimposed in *c* and *f*. (*c*) Apparently uninjured axons contain long and intact NF200 positive filaments (arrowed), with no SAP staining. (*a*′–*c*′, *d*′–*f*′) Intense SAP immunostaining (arrows) of damaged axons colocalized with NF200 immunostained, swollen neurofilaments and spheroids (arrowheads). (*d*″–*f*″) Strong SAP immunostaining in neuronal cell body containing some spheroids. (*a*′–*c*′, *d*′–*f*′) Enlargements of dashed box regions of corresponding images. Scale bars: *a–f*, 100 µm; *a*′–*f*′& *d*″–*f*″, 25 µm. Some images include blue nuclear counterstaining with Hoechst.

In damaged axons, cytoskeletal neurofilaments swell and form spheroids [49,50] and in damaged neurones they form cytoplasmic inclusion bodies [51]. Double immunostaining for SAP and the neurofilament heavy chain marker, NF200, demonstrated that SAP deposition was particularly associated with these signs of neuronal and axonal damage (figure 4*a–f*). By contrast, anti-SAP antibody did not stain apparently uninjured axons in which anti-NF200 antibodies stained long and intact neurofilaments (figure 4*c*).

### 3.5. Early and persistent intra-cerebral SAP deposition after TBI

**Figure 5.**
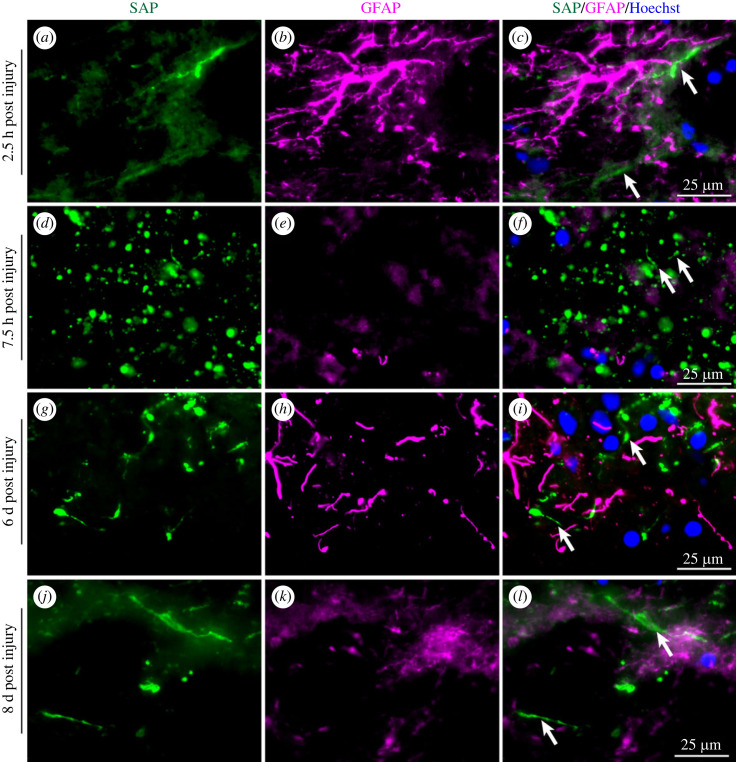
Immunostaining for SAP in brain persists after TBI. Single sections of brain obtained at various times after TBI were double immunostained with anti-SAP (green, arrows) and anti-GFAP (magenta). (*a–c*) 2.5 h, (*d–f*) 7.5 h, (*g–i*) 6 h, (*j–l*) 8 d after injury. Scale bars: 25 µm. Some images include blue nuclear counterstaining with Hoechst.

**Figure 6.**
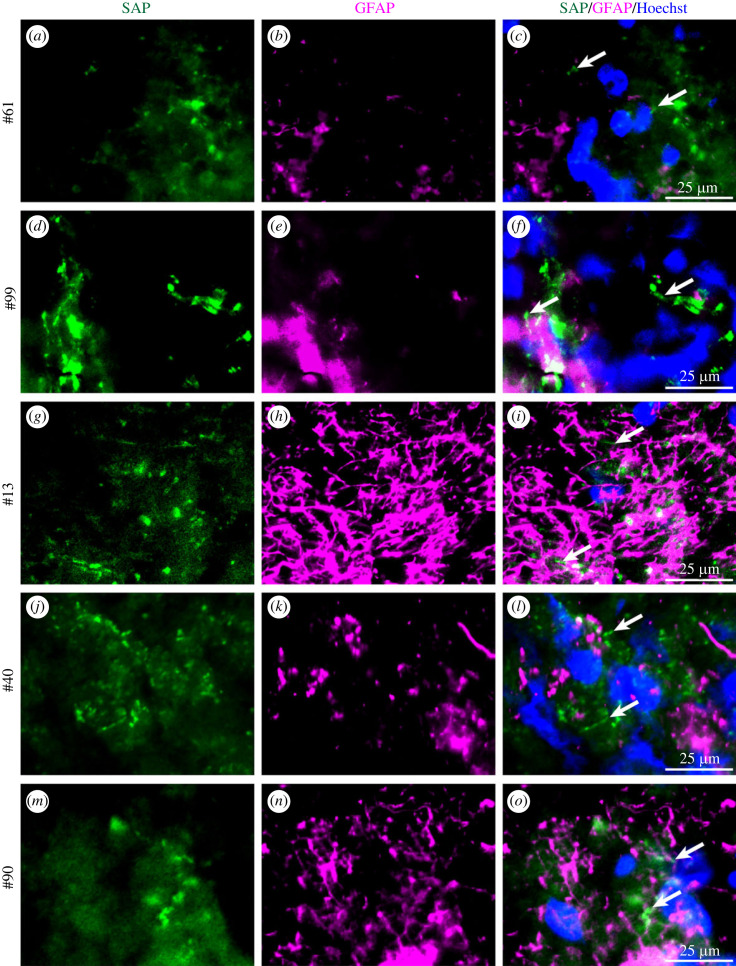
SAP immunostaining of contused brain tissue from an independent severe TBI patient cohort in Taiwan. Single sections of brain obtained at various times after TBI were double immunostained with anti-SAP and anti-GFAP. (*a–o*) Sections of surgically resected tissue from five different patients (individual case numbers on left) immunostained for SAP (green, arrows) and GFAP (magenta). Scale bars: 25 µm. Some images include blue nuclear counterstaining with Hoechst.

Immunostaining for SAP was already present in contused tissue 2.5 h after injury (figure 5*a–c*), in the earliest sample available. Staining for SAP was also positive in samples taken at 7.5 h, 6 days and 8 days after TBI (figure 5*d–l*). Identical anti-SAP staining was also observed in contused brain tissues from the five severe TBI cases studied independently in Taiwan (figure 6*a–o*), obtained between 2 h and 42 h after the injury, confirming the early appearance and prolonged duration of SAP deposition on damaged neurones and their processes.

## 4. Discussion

SAP promotes the formation and persistence of amyloid fibrils, including A*β* type [30–33]. Unrelated to amyloid, SAP has been shown in experimental studies *in vitro* and *in vivo* to be directly neurotoxic for some cerebral neurones [36–39,52] SAP might thus contribute to pathogenesis of neurodegeneration by these two distinct mechanisms. In support of the direct neurotoxic effect, a preliminary report showed a closer relationship of cognition with brain SAP content than with typical Alzheimer's disease neuropathology [53]. This was confirmed and extended by the recent, much larger cognitive function and ageing study of neocortical SAP content and dementia status at death in the elderly [40] Neocortical SAP content was significantly higher in individuals who were demented at death than in those without dementia, such that individuals in the top tertile of neocortical SAP content had a five-fold greater odds ratio of being demented at death. Furthermore, the relationship between SAP content and dementia was independent of all classical neuropathological scores. SAP content was thus not just a surrogate marker for the amyloid and tau neuropathology known to be associated with dementia. The independent association of brain SAP content with dementia is consistent with a direct neurotoxic effect.

Here we have extended to TBI, which is a prevalent unmet medical need, the potential association between increased brain exposure to SAP and cognitive loss. The BBB is frequently breached after TBI, both by direct trauma and more subtly [54,55], but access of circulating SAP to the brain, where the normal SAP concentration in the CSF is one thousand-fold lower than in the plasma [25,26], has not previously been investigated. We have confirmed that SAP rapidly enters damaged brain tissue, is deposited specifically on and in damaged neurones and their processes, notably associated with the cytoskeletal neurofilaments, and persists for at least several days. These observed associations obviously cannot establish causality and the study was neither designed for, nor capable of, determining whether SAP deposition caused neuronal damage or simply reflected passive binding to already damaged cells. Nevertheless, the specificity of binding only to some neurones, to their neurofilament cytoskeleton, and not at all to glial or microvascular endothelial cells, is consistent with a potential pathogenetic role.

The specificity of SAP deposition was demonstrated by the fact that there was no comparable deposition of CRP in the same sections. CRP and SAP are closely related, with very similar structure and size, and overlapping plasma concentrations [46]. However, crucially, they differ in their precise calcium dependent ligand binding specificities, which underly all the well validated and reproducible biological functions of both proteins, including the *in vitro* neurotoxicity of SAP [56].

Our novel findings in brain after TBI raise the important question of whether the unpredictable, but potentially greatly increased, cerebral exposure to neurotoxic and pro-amyloidogenic SAP, might contribute to the also unpredictable, medium and long-term neuropathological, neurodegenerative and cognitive sequelae. Fortunately, this question can be addressed using the experimental drug, miridesap (hexanoyl bis-D-proline; (R)-1-[6-[(R)-2-carboxy-pyrrolidin-1-yl]-6-oxo-hexanoyl]pyrrolidine-2-carboxylic acid). Miridesap, which is safe, well tolerated, and free of significant side or adverse effects, profoundly depletes circulating SAP for as long as the drug is administered [57,58] and removes all SAP from the CSF [26] and from the brain [59]. Miridesap is currently being trialled in DESPIAD (EudraCT number 2016-003284-19), an academic, UK National Institute for Health Research funded, phase 2b study of SAP depletion in AD, which will report in 2025. Regardless of the trial outcome, the opportunity for an accelerated trial in TBI is attractive. In contrast to the potential roles of SAP in neurodegeneration and amyloidogenesis during evolution of AD neuropathology and cognitive loss over decades, the precise onset of SAP overexposure is known in TBI and the progression of neuropathology, including A*β* amyloid deposition, and of cognitive loss, are greatly accelerated compared to AD. If SAP is a significant pathogenic factor, its immediate depletion from blood and brain by miridesap, should therefore deliver a relatively prompt outcome.

## Data Availability

The data supporting the reported results are available within the article. The original images displayed are available from the corresponding author, upon reasonable request. Supplementary material is available online [60].
